# Dyskeratosis congenita: rare case report of Syria

**DOI:** 10.1093/omcr/omab041

**Published:** 2021-11-25

**Authors:** Firas Hussein, Zainab Omar

**Affiliations:** Department of Clinical Hematology, Tishreen University Hospital, Tishreen Street, Lattakia 041, Syria

**Keywords:** High-digestive endoscopy, Esophageal varices

## Abstract

Dyskeratosis congenita (DC) is an inherited disease characterized by the triad of abnormal skin pigmentation, nail dystrophy and mucosal leukoplakia. Non-cutaneous abnormalities (dental, gastrointestinal, genitourinary, neurological, ophthalmic, pulmonary and skeletal) have also been reported. Bone marrow failure (BMF) is the main cause of early mortality, with an additional predisposition to malignancy. DC results from an anomalous progressive shortening of telomeres resulting in DNA replication problems inducing replicative senescence. Men are more affected than women are and X-linked recessive, autosomal dominant and autosomal recessive forms of the disease are recognized. There are no targeted therapies for DC. Patients treated with androgens had a hematological response. We herein describe case of a 32-year-old man, presented with several characteristic systemic features of this condition, including the classic triad of lesions, dysplastic bone marrow, epiphora and liver cirrhosis with grade I esophageal varices. Therefore, a prophylactic propranolol was started in additional to danazol. Three-week later, the patient had subsequent increases in his platelet, red cell and white cell counts.

## INTRODUCTION

Dyskeratosis congenita (DC; also called Zinsser Engman-Cole syndrome or short telomere disease) is a rare inherited genetic condition presenting with multisystem involvement. It was firstly reported by Zinsser in 1906, and then by Engmann in 1926 and Cole in 1930 [1]. The prevalence of classic DC is ~1/1 000 000 individual. The syndrome shows a typical triad of oral leukoplakia, nail dystrophy and reticular hyperpigmentation. It is frequently complicated by malignancy and bone marrow failure. Males are more affected than females since the inheritance is classically x-linked recessive and most cases occur between the ages of 5 and 10 years [2]. We report a case of 32-year-old patient with DC in our department of clinical hematology at Tishreen University Hospital, in an attempt to increase awareness about this rare disease.

## CASE REPORT

**Figure 1:**
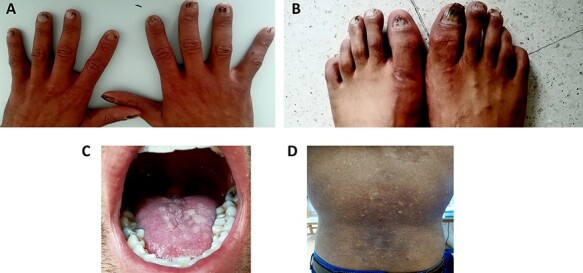
Nail dystrophy (**a**,**b**); Leukokeratosis plaques on the tongue (**c**); Abnormal skin pigmentation of back area (**d**).

**Figure 2:**
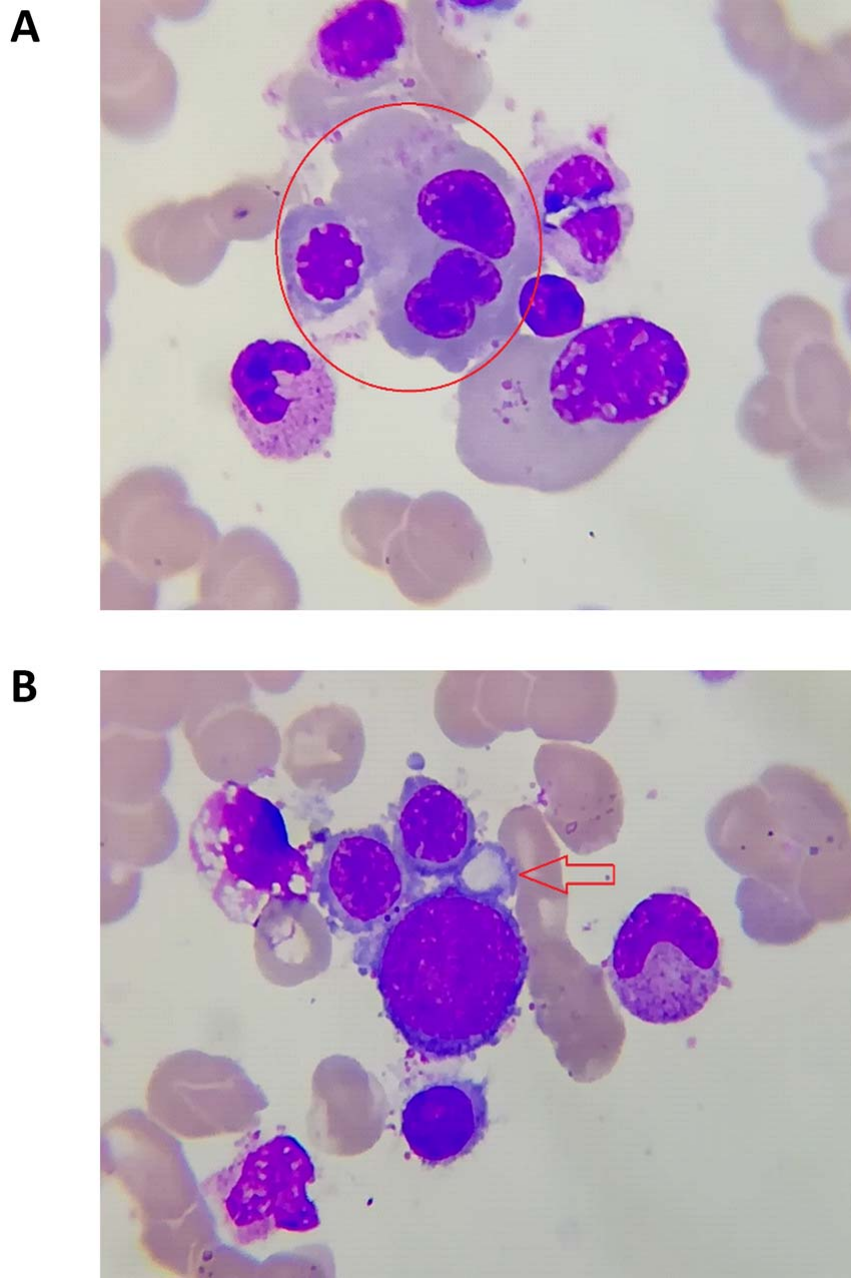
Dysplastic features of erythroid linage.

A 32-year-old male, without significant past medical history, he was admitted in the department of clinical hematology for a diagnostic workup for fatigue and dyspnea on exertion. At admission, patient appeared pale but vital signs were within normal limits. Physical examination revealed nail dystrophy (Fig. 1a and b), leukokeratosis plaques on the tongue (Fig. 1c), Hyperkeratotic and pigmented patches were present on the back, neck, trunk and palms (Fig. 1d). Patient did not have a history of tobacco usage. The onset of the cutaneous lesions was at 7 years of age with slow and progressive accentuation. Patient mentioned presence of similar case in his family (brother, uncle {maternal side}). Mental retardation was not observed. Admission labs included WBC: 2.200 cells/ul, Absolute neutrophil count: 1.500 cells/ul, hemoglobin 8 g/dl, platelet: 65 000/ul, creatinine: 1 mg/dl, ALT: 13 U/L, international normalization ratio (INR):1.9. Bone marrow aspiration and biopsy was done and showed normocellularity with trilineage dysplastic changes, dysplastic changes in RBC lineage were characteristic (Fig. 2a and b). According to these findings, the diagnosis of myelodysplastic syndrome (MDS) was confirmed. Ultrasound (US) of the abdomen revealed, coarsened hepatic echotexture, moderate splenomegaly 15.5 cm and an important dilation of the splenic veins at the splenic hilum and the portal veins. Therefore, portal hypertension was suspected due to hepatic cirrhosis/fibrosis. The patient was referred for gastrointestinal consultation then prepared for high-digestive endoscopy, which revealed, grade I esophageal varices. Unfortunately, percutaneous liver biopsy was contraindicated due to tendency to bleed because of thrombocytopenia and prolonged INR. Therefore, prophylactic propranolol was initiated. Viral marker for hepatitis B or C that could cause pancytopenia showed no specific viral infection. The patient did not present with respiratory complaint, chest X-ray indicated hilar enlargement after which CT of the chest showed no abnormality. Epiphora was mentioned and punctual atresia was suspected but the patient refused to be referred for ophthalmologic consultation. The patient in this study presented all the components of the triad, in addition to bone marrow dysplasia, hepatic cirrhosis and ophthalmologic manifestation. We started the treatment with danazol a synthetic androgen derivative also known as 17α-ethinyl testosterone. Three-week later a good hematologic response was observed, WBC: 5500 cells/ul, Absolute neutrophil count: 3000 cells/ul, Hgb: 9 g/dl and Plt: 95 000/ul. The patient currently undergoing outpatient treatment at the hematology and gastroenterology outpatient clinics.

## DISCUSSION

DC is a rare disease with an estimated annual incidence of one case in one million, with multiple and variable clinical manifestations, the classic and initial form are usually characterized by the mucocutaneous triad of abnormal skin pigmentation, nail dystrophy and leukoplakia [3]. Patients with DC have been shown to have disease diversity in terms of age at onset, symptoms and severity. Even if the patients have the same gene mutation, the manifestations of the disease are variable, which is why sometimes it is difficult to make a correct diagnosis [4]. Nail changes are characterized by thin, dystrophic nails that may be markedly shortened and fragile with a subungual thickening, the dystrophy usually appears in the first decade of life and begins as longitudinal ridging and splitting. With the progression of the disease, it eventually leads to pterygium formation with the distal expansion of the hyponychium and obliteration of the distal groove [5]. Skin findings can be variable, ranging from tan-to-gray macular or patchy areas of hyper or hypopigmentation with a reticulated or mottled pattern. Atrophy and telangiectasia can also be present (poikiloderma), DC is also frequently associated with oral findings, which include oral leukoplakia, increased dental caries, hypodontia, thin enamel structure, aggressive periodontitis, intraoral brown pigmentation, tooth loss, taurodontism and blunted roots [6]. The patient described in this paper showed reticulate hyperpigmentation of the skin with onset in the seventh year of life, nail dystrophy on the toes and leukokeratosis plaques on the tongue—that is, the three components of the classic triad. The BM findings in DC are variable and range from normal to different severity of aplasia depending on the stage of the disease. Sometimes it is indistinguishable from aplastic anemia due to other causes [7]. The BM abnormalities can progress in different forms with the appearance of myelo-dysplasia in one or more lineages or leukemia [3]. In our 32-year-old patient, the initial labs revealed pancytopenia. Then, MDS was confirmed according to bone marrow aspiration and biopsy findings. Hepatic complications have been described, too. Some studies reported that loss-of-function telomerase gene variants are risk factors for sporadic cirrhosis. However, there is not a definitive conclusion that can explain the appearance of hepatic complications in DC. The possible hepatic complications can show diverse histological liver features such as necrosis, fibrosis, cirrhosis, inflammation and hyperplasia, although hepatic lesions are not a frequent complication in DC (~7%) [8]. In our case, US of the abdomen suggested portal hypertension and liver cirrhosis. Then, high-digestive endoscopy confirmed the presence of grade I esophageal varices. Therefore, prophylactic propranolol was started. Pulmonary fibrosis characterized by loss of lung epithelium, prominent fibrosis and impaired gas exchange [9]. In addition, abnormalities of pulmonary vasculature are seen in ~80% of cases [9]. Our patient had no pulmonary manifestation. The clinical diagnosis of DC is based on the presence of the four major features of the disease, which include the mucocutaneous triad and bone marrow failure, as well as the presence of multisystem features of the disease: epiphora, developmental delay or mental retardation, pulmonary disease, periodontal disease, esophageal stricture, premature hair graying or loss, hyperhidrosis or development of malignant lesions. To make a correct clinical diagnosis of DC requires at least two of four major features and at least two multisystem features, and sometimes it is difficult because of the disease heterogeneity [3]. Our patient had the mucocutaneous triad, in additional to MDS, hepatic involvement and ophthalmic manifestation. There is no effective and curative treatment for DC. It is now apparent that androgens can elicit a hematologic response in a substantial proportion of patients with specific inherited BMF syndromes, namely Fanconi anemia and DC [10]. In our case, the patient began taking danazol and 3 weeks later a good hematologic response was detected. We experienced a patient who was diagnosed as DC with MDS. DC is being now studied ever more intensively and this case report can be beneficial not only to the health care professional but to those suffering from this rare disease.

## FUNDING

No funding was received.

## CONFLICT OF INTEREST STATEMENT

No conflict of interest.

## ETHICAL APPROVAL

Written consent was obtained from the patient to participate in the study.

## CONSENT

Written consent to publish this information was obtained from patient.
